# Complications and nutrient deficiencies two years after sleeve gastrectomy

**DOI:** 10.1186/1471-2482-12-13

**Published:** 2012-07-05

**Authors:** Nicole Pech, Frank Meyer, Hans Lippert, Thomas Manger, Christine Stroh

**Affiliations:** 1 Department of General, Abdominal and Pediatric Surgery, Municipal Hospital Gera, Strasse des Friedens 122, Gera, 07548, Germany; 2 Department of General, Abdominal and Vascular Surgery, University Hospital, Magdeburg, Germany

**Keywords:** Sleeve gastrectomy, Laparoscopic sleeve gastrectomy, Obesity, Metabolic surgery, Bariatric surgery, Nutritional deficiencies

## Abstract

**Background:**

The aim of this systematic study was to investigate patient outcomes and nutritional deficiencies following sleeve gastrectomy (SG) during a median follow-up of two years.

**Methods:**

Over a period of 56 months, all consecutive patients who underwent SG were documented in this prospective, single-center, observational study. The study endpoints included complication rates, nutritional deficiencies and percentage of excess weight loss (%EWL).

**Results:**

From September 26, 2005 to May 28, 2009, 100 patients (female: male = 59:41) with a mean age of 43.6 years (range: 22–64) and a preoperative BMI of 52.3 kg/² (range: 36–77) underwent SG. The mean operative time was 86.4 min (range: 35–275). Major complications were observed in 8.0 % of the patients. During the follow-up period, 25 patients (25.0 %) underwent a second bariatric intervention (22 DS and 3 RYGBP). Out of the total 100 patients, 48 % were supplemented with iron, 33 % with zinc, 34 % with a combination of calcium carbonate and cholecalciferol, 24 % with vitamin D, 42 % with vitamin B12 and 40 % with folic acid. The patients who received only a SG (n = 75) had %EWL of 53.6, 65.8 and 62.6 % after 6, 12 and 24 months, respectively.

**Conclusions:**

SG is a highly effective bariatric intervention for morbidly obese patients. Nutritional deficiencies resulting from the procedure can be detected by routine nutritional screening. Results of the study show that Vitamin B12 supplementation should suggested routinely.

## Background

Obesity has developed into an epidemic. Approximately 1.7 billion people are overweight, and 312 million are obese [1,2]. In Germany in 2009, 60.1 % of male and 42.9 % of female population was overweight [3]. There are currently no conservative treatments that produce the %EWL results and stable courses observed following bariatric surgery. Obesity is associated with an increased mortality risk [4]. Obesity is also associated with increased health costs. A BMI = 35 kg/² is associated with a 200 % increase in health care costs compared the normal weight range [5].

As a result of the obesity epidemic bariatric and metabolic surgeries have grown in popularity in recent years, resulting that the number of operations is rapidly increasing. Laparoscopic sleeve Gastrectomy (SG) was performed as the single step procedure for surgically induced weight loss in 2000 [6].

SG can be suggested as a first step procedure for multimorbid patients with a BMI > 50 kg/², considering the high mortality rate of 6 % following biliopancreatic diversion (BPD) with DS [7,8]. In literature is the lack of studies with high evidence levels on SG reporting long term follow up data, results on reoperation rate or long term complication rate for surgical complications as well as nutrient deficiencies.

The aim of the following systematic study was to investigate nutritional deficiencies and outcomes following SG during a mean follow up period of two years.

## Methods

From September 26, 2005 to May 28, 2009, 100 patients underwent SG in the Surgery Department of the SRH Wald-Klinikum Gera Hospital. All patients had to agree with an informed consent. Data collection and analysis was performed in compliance with the Helsinki Declaration.

After we ensured compliance with international and German guidelines all patients had to take part in an informational seminar [9]. Patient´s evaluation was performed by experienced bariatric surgeons.

Data collection took place prospectively and analyzed retrospectively. Patients were classified according to the WHO classifications of obesity (35–39.9 kg/²; 40–49.9 kg/²) with expansions to “super obesity” (50–59.9 kg/²) and “super-super obesity” (= 60 kg/²). Analyzed parameters are listed in Table 1 (Table 1). Acute and postoperative complications were evaluated.

**Table 1:** Recorded parameters

**Age**	**OP duration**
Sex	Use of staple line reinforcement
Length of hospital stay	Bougie size
Type of Operation	Resected gastric volume
Laboratory parameters	
Iron	Albumin
Zinc	Vitamin B12
Selenium	Folic acid
Alkaline phosphatase	Calcium
Hemoglobin	Parathyroid hormone

### Sleeve gastrectomy- operation technique

SG was performed in the French position in a 30° reverse Trendelenburg position. Pneumoperitoneum was established to 15 mmHg. First trocar for placing the camera was inserted 15 cm distal to the xiphoid process. Another trocar was placed on the epigastric angle for liver retraction. Two trocars were located on the right and left upper quadrants. A bougie 31–36 French was used. The dissection of the greater curve began 5–6 cm proximal to the pylorus and extended to the angle of His. Sleeve resection of the stomach was performed using an Endo GIA stapler (green) made by Covidien, Germany® using staple line reinforcement in 88 % of the patients. Staple line was not oversewn. To exclude leakage of staple line a methylene blue test was performed. The resected stomach was filled with water to determine the resected gastric volume. Histopathological analysis was performed on the specimen. In all patients for single shot antibiosis a third generation cephalosporine was given.

### Postoperative follow up

All of the patients were examined throughout a 24-month follow-up period (at 3, 6, 12, 18 and 24 months postoperatively) in our clinical outpatient department. Furthermore, short- and long-term results with regard to BMI, weight, %EWL and important laboratory parameters (iron, zinc, selenium, alkaline phosphatase, hemoglobin, MCV, albumin, vitamin B12, folic acid, calcium and parathyroid hormone levels) were registered (Table 1).

## Results

### Demographic data

From September 26, 2005 to May 28, 2009, 100 patients (sex ratio, females: males = 59:41 [1.4:1]) with a mean age of 43.6 years (range, 22–64) and a preoperative BMI of 52.3 kg/m² (range, 36–77) underwent SG. Operation was performed by three surgeons, operating as a team in all the 100 recorded operations. Patient´s outcome and operation time were not influenced by changing the surgeon in these team. Demographic data are shown in Table 2 (Table 2).

**Table 2:** Data from patients and operations

		**BMI**				**Total**
	**[kg/²]**	**35-39,9**	**40-49,9**	**50-59,9**	**> 60**	
Sex						
Male	[%]	62.5	24.1	46.3	45.5	41.0
Female	[%]	37.5	75.9	53.7	54.5	59.0
Total	[n]	8	29	41	22	100
Mean Age Age range	[years]	44.6 33-64	44.3 22-59	43.8 22-58	41.7 25-59	43.6 22-64
Mean BMI BMI range	[kg/²]	38.3 36.0-39.5	44.4 40.0-49.8	53.5 50.0-59.1	65.5 60.0-77.0	52.3 36.0-77.0
Operation						
Laparoscopy	[%]	100.0	96.6	95,1	81.8	93.0
Laparoscopy with conversion	[%]		3.4	2,4	18.2	6.0
Laparotomy	[%]			2,4		1.0
Mean Operative time Operative time range	[min]	70.4 41-101	70.2 44-120	92.9 35-275	101.2 45-225	86.4 35-275
Mean Resected gastric volume	[ml]	785.7 (650–900)	882.4 (600–1200)	1081.8 (500–1700)	1045.5 (700–1700)	995.6 (500–1700)
Bougie size						
31 French			3.4			1.0
32 French	[%]	12.5	3.4	7,3	9.1	7.0
34 French	[%]	75.0	93.1	87,8	90.9	89.0
36 French	[%]	12.5		4,9		3.0
Staple line reinforcement	[%]	87.5	96.6	85,4	81,8	88.0

#### Surgical outcome

##### Operation data

Of the 100 patients, 99 underwent primarily laparoscopic surgery. In 6.1 % of these patients (6 of 99), a conversion from laparoscopy to laparotomy was necessary. In one case, a primary laparotomy was performed because of an abdominal wall hernia, resection of an anus praeter, subtotal colectomy with an ileorectostomy. Subtotal colectomy was performed due to the fact of several colon operations in an outside hospital. Postoperative course of the patient was uneventful. In 4 cases, this conversion was performed because of an insufficient laparoscopic overview with high intraabdominal pressure and in 2 cases due to the fact of laparoscopically uncontrollable bleeding (Table 3).

**Table 3:** Acute and postoperative complications

**Complications (20/100; 20.0 %)**			
**Cause of acute complications / conversions**		**Postoperative complications**	
	[n]		[n]
Insufficient intraabdominal view	4	Leakage	3
Insufficient intraabdominal view Bleeding	2	Abscess	5
		Severe sepsis	2
		Perforation of duodenum	1
		Pleural effusion	2
		Pneumonia	3
		Thrombosis	1
		Wound infection	6
		Death	1

The mean operation time was 86.4 min. The mean resected gastric volume was 995.6 ml. A 34-French calibration tube was used in 89 % of the patients (89). Staple line reinforcements were used in 88 % of the patients (88) (Table 2). Comparing leakage rate and bleeding in patients using staple line reinforcement or oversewing was no difference.

There were significant differences among the durations of the OP. When staple line reinforcements were used, the mean OP duration was 79.3 min, compared to 141.1 min without using staple line reinforcements (*p* = 0.010).

A conversion to laparotomy was significantly more necessary for patients with a BMI > 60 kg/² compared to patients with a lower BMI (*p* > 0.001). The duration of the OP averaged 70.4 min for patients with a BMI between 35 and 39.9 kg/², 70.2 min for patients with a BMI between 40 and 49.9 kg/², 92.9 min for patients with a BMI between 50 and 59.9 kg/² and 101.2 min for patients with a BMI > 60 kg/². Patients with a BMI > 50 kg/² had significantly longer OP durations compared to patients with a BMI < 50 kg/² (95.7 vs. 70.2; p = 0.001). The resected gastric volume was significantly higher in patients with BMI > 50 kg/² compared to those with BMI < 50 kg/² (1072.7 vs. 854.2; *p* = 0.001).

##### Intraoperative and early postoperative surgical complications

Twenty patients (20.0 %) suffered on intraoperative or/and postoperative complications (Table 3). Postoperative complications occurred in 17 patients (17.0 %). One patient with BMI 55.5 kg/² died (1.0 %). At the tenth postoperative day patient complained of left upper abdominal pain. The CT scan showed an insufficient suture with a subcardial abscess. A CT-guided puncture ensued. Patient’s cardiac situation worsened and ARDS developed. Acute complications were observed significantly more frequently in patients with BMI > 60 kg/² (p < 0.001). The major complication rate was 8 % (Table 3).

##### Mortality rate

Mortality rate after 24 month of total follow up is 2 %. Above mentioned patient died during hospital stay 73 days after operation, due to SIRS and ARDS. Second patient died several months after SG in fact of his cardiac situation without any relation to operation.

##### Follow up data

Follow up rate was 80 % (80/100). All of these patients were clinical examined with a laboratory test 24 months after SG, so mean follow up time is 24 months.

The mean preoperative BMI of all of the patients examined was 52.3 kg/². At the end of the follow up, there was a significant reduction in BMI to 35.4 kg/² (*p* < 0.0005). The greatest weight loss occurred within the first 12 postoperative months (52.3 kg/² to 36.3 kg/²). Afterwards we observed a weight loss from 36.3 kg/² to 35.4 kg/² for all of the patients.

The %EWL in the BMI categories between 35 and 39.9 kg/² and 40 and 49.9 kg/² was 47.4 % and 47.5 %, respectively, after 3 months. The greatest %EWL in these categories was achieved after 12 (72.6 %) and 24 (74.2 %) months. Patient´s with a BMI between 35 and 39.9 kg/² showed a slight tendency toward increased weight after this time. Patient´s with a BMI between 40 and 49.9 kg/², 50 and 59.9 kg/² and over 60 kg/² showed continuous weight loss throughout the entire 24-month follow-up period (Table 4). On average, there was a tendency toward increased weight after 18 months. The most significant weight loss was achieved within the first postoperative year (*p* < 0.0005). Regarding the percentage overweight loss, the highest %EWL of 67.1 % occurred after 18 months, and after 24 months, there was a further %EWL of 62.6 %. The highest %EWL of 83.3 % was observed in patients with a BMI between 35 and 39.9 kg/² after 12 and 18 months (Table 4).

**Table 4:** **Weight progression (BMI in kg/²) of patients without a second operation during the follow-up period (n = 75) and** %**EWL of patients without a second operation during the follow-up period**

**months BMI**	**OP**	**3**	**6**	**12**	**18**	**24**
35-39.9 kg/²	38.2	31.0	30.2	27.4	27.5	28.4
40-49.9 kg/²	44.2	35.0	31.1	29.4	30.0	29.9
50-59.9 kg/²	53.2	44.0	39.5	37.6	36.9	38.2
≥60 kg/²	65.6	52.5	48.1	44.0	42.3	43.7
Total	51,0	40.8	38.4	34.4	34.4	35.6
%EWL						
35-39,9 kg/²		53.4	62.5	83.3	83.3	76.3
40-49,9 kg/²		48.6	67.4	76.4	75.0	74.5
50-59,9 kg/²		33.6	48.5	54.7	57.3	52.8
≥60 kg/²		30.3	42.6	52.5	58.6	53.8
Total		40.7	53.6	65.8	67.1	62.6

##### Revisional procedures after SG

Over the total observation period of 24 months, a second operation to induce weight loss was required in 25.0 % (25) of the patients to develop further weight loss or amelioration on comorbidities. Three patients underwent RYGBP and 22 patients DS.

#### Nutrient deficiencies, laboratory parameters and supplementation

In patients after SG as a single step procedure a postoperative routine supplementation was not performed. Supplementation was suggested according laboratory examination performed every 6 months in case of deficiencies.

##### Iron

Iron supplementation was performed in 48 patients (48.0 %). Seven of these patients developed microcytic anemia, which required the initiation of iron supplementation. In 23 of these 48 patients, iron supplementation was performed as prophylaxis after RYGBP or DS. The other 25 patients (25.0 %) 21 of them female were supplemented after SG. Further we examined iron supplementation in fertile woman. Women had a mean age of 42.8 years (25–59). Thirteen of these 21 women recorded a reduced iron value, and the other 8 women were supplemented with a combination of folic acid and iron.

##### Zinc

The highest average value for zinc of 14.70 μmol/L was determined preoperatively (reference range: 10–23 μmol/L). There were no significant differences among the average values in the follow-up period. In total, 33 patients underwent zinc supplementation, and 5 of these complained of hair loss. Nineteen patients were supplemented after RYGBP or DS. Fourteen patients (14.0 %) were supplemented following SG due to zinc deficiency. For supplementation patients were given 15 mg Zink daily.

##### Selenium

The highest average value for selenium of 81.60 μg/L (reference range: 50–120 μg/L) was determined preoperatively. After 3 months, a significant decrease to 61.13 μg/L (*p* < 0.0005) occurred. No other significant differences were observed over the course of the follow-up. Due to selenium deficiency in laboratory eight patients after SG were treated with selenium supplementation using 100 μg twice a day. Among the 75 patients who did not undergo a second operation, there was a gradual increase in the concentration of selenium (OP: 81.5 μg/L; 3^rd^ month: 62.1 μg/L; 6^th^ month: 63.0 μg/L; 12^th^ month: 66.9 μg/L; 18^th^ month: 66.8 μg/L; 24^th^ month: 69.7 μg/L). The increase in selenium from 3 months after the operation achieved a significant level after 12 months (*p* = 0.043).

##### Calcium and parathyroid hormone

In 62 of the 100 patients, PTH levels were preoperatively determined, and 22.6 % of the patients (14) had hyperparathyroidism. The average PTH levels (reference range: 10.0-69.0 ng/L) for patients with BMIs over 60 kg/² were 83.15 ng/L preoperatively, 73.30 ng/L after 6 months and 61.55 ng/L after 18 months (Table 5; 6). Thirty-four patients (34.0 %) were supplemented with calcium carbonate and cholecalciferol, including 15 patients supplemented after RYGBP or DS. For calcium supplementation patients were supplemented with 500 mg calcium with 10 mg cholecalciferol four times daily. Twenty-four patients (24.0 %) were treated with separate or additional vitamin D supplementation due to high levels of PTH, including 9 patients treated preventively after a second operation.

**Table 5:** Postoperative course of calcium (mmol/l)

**Timeline [months] BMI [kg/²]**	**OP**	**3**	**6**	**12**	**18**	**24**
35 - 39,9	2,36	2,36	2,37	2,40	2,40	2,44
40 - 49,9	2,38	2,37	2,40	2,35	2,38	2,36
50 - 59,9	2,37	2,35	2,38	2,34	2,35	2,30
≥ 60	2,33	2,44	2,40	2,34	2,35	2,33
Total	2,36	2,37	2,39	2,35	2,36	2,34

Under supplementation, a rising concentration of PTH appeared 3 months after the operation. After 6 months, a significant decrease in the concentration of PTH was identified (*p* = 0.045). Course of PTH levels is shown in table 6 (Table 6).

**Table 6:** Postoperative course of parathormone (ng/l)

**Timeline [months] BMI [kg/²]**	**OP**	**3**	**6**	**12**	**18**	**24**
35 - 39,9	44,93	45,45	41,98	42,04	44,70	39,90
40 - 49,9	57,34	65,65	51,73	54,23	56,01	54,94
50 - 59,9	48,98	46,93	53,29	55,95	55,79	56,28
≥ 60	83,15	108,82	73,30	60,41	61,55	64,81
Total	59,55	66,67	56,72	55,13	56,44	56,52

##### Albumin

SG did not significantly affect the patients’ albumin levels (reference range: 34.0-48.0 g/L) during the follow-up period.

##### Vitamin B12

Overall, forty-two patients (42.0 %) received vitamin B12 supplementation. For vitamin B12 supplementation 1000 μg Vitamin B12 monthly was ordinated. 24 patients with SG as a standalone procedure (24.0 %) were supplemented within the first postoperative year and 18 patients after RYGBP or DS. (Table 7). Under supplementation, the vitamin B12 levels achieved stable average values (reference range: 175–810 pmol/L) during the entire follow-up period.

The 75 patients after SG as a standalone procedure demonstrated stable and not significantly different vitamin B12 concentrations (OP: 285.6 pmol/L; 3^rd^ month: 288.1 pmol/L; 6^th^ month: 269.0 pmol/L; 12^th^ month: 253.8 pmol/L; 18^th^ month: 254.2 pmol/L; 24^th^ month: 265.2 pmol/L) (Table 7).

**Table 7:** Necessity of vitamin B12 supplementation during the follow-up period

	**BMI**									
	**35-39.9 kg/²**		**40-49.9 kg/²**		**50-59,9 kg/²**		**≥ 60 kg/²**		**Total**	
Vitamin B12	[n]	[%]	[n]	%	[n]	[%]	[n]	[%]	[n]	[%]
After OP	0	0.0	0	0.0	0	0.0	1	4.5	1	1.0
After 3 months	1	12.5	4	13.8	5	12.2	2	9.1	12	12.0
After 6 months	0	0.0	2	6.9	4	9.8	2	9.1	8	8.0
After 12 months	0	0.0	1	3.4	0	0.0	2	9.1	3	3.0
After 24 months	0	0.0	0	0.0	0	0.0	0	0.0	0	0.0
After 2nd OP	1	12.5	2	6.9	10	24.4	5	22.7	18	18.0
Total	2	25.0	9	31.0	19	46.3	12	54.5	42	42.0

##### Folic acid

Regarding folic acid (reference range: 10.40-42.40 nmol/L), there was a significant decrease 3 months after the operation from 18.87 nmol/L to 15.29 nmol/L (*p* < 0.0005). 19 patients were supplemented. After RYGBP or DS 21 patient were given a supplementation according national and international guidelines. After the third month following the operation, an increasing concentration of folic acid was observed with a maximum average of 20.96 nmol/L after 24 months. Supplementation was performed with a combination of folic acid 0.5 mg and iron 40 mg daily.

## Discussion

SG is an effective operative method for inducing weight loss. SG can be performed as the first step of a two-stage procedure for high-risk patients to reduce the perioperative risks of DS or RYGBP.

Literature shows the benefits of LSG compared to laparoscopic gastric banding (LAGB) and laparoscopic RYGBP. Advantages of SG are non-resection of the pylorus, which prevents dumping syndrome; no intestinal anastomoses, no risk of developing an internal hernia and nearly regular intestinal absorption [10]. Complication rate of SG procedure is still high, especially short term complications as leakage and staple line insufficiency influences the complication rate. In literature an increasing long term complication rate is reported due to stenosis, gastroesophageal reflux and re-operation rate due insufficient weight loss, regain of weight or insufficient amelioration of comorbidities [11]. Evidence based data on nutrient deficiencies, especially vitamin B12 and iron, after SG is not available.

SG, however, reduces perioperative risks of morbidly obese patients with BMI > 60 kg/² as a first step procedure [12]. The reported initial weight loss after SG spans a wide range, between 33 and 83 % [13,14]. In a prospective study of 100 patients, Johnston et al. presented a %EWL of 60 % after 5 years [15]. That study group achieved a %EWL of 60.3 % after 12 months and 63.8 % after 24 months.

Over a 24-month period, the entire patient population experienced continuous weight loss. The weight loss remained constant (BMI 35.4 kg/²) in clinical examinations through the 24^th^ months. SG as a single step operation is suitable for patients with BMIs < 50 kg/². Only 8.1 % of these patients (3/37) required a second intervention to induce further weight loss within the follow-up period (vs. 34.9 % with BMI of 50 kg/²). After 24 months, patients with a BMI between 35 to 39.9 kg/² achieved the highest %EWL. Therefore, there was no correlation between the resected volume of the stomach and the %EWL. Only one patient (12.5 %) needed to undergo a second operation for further weight loss.

After 18 months, patients who only underwent SG demonstrated increased mean weights, which may have been due to sleeve dilatation. This possibility was considered by Gluck et al., who presented %EWLs of 67.9 % after 1 year, 62.4 % after 2 years and 62.2 % after 3 years for patients after SG with preoperative BMIs between 35 and 43 kg/² [16].

There is not always sufficient weight loss after SG; insufficient changes in food patterns or potential recidivism to old food patterns may cause a sleeve dilatation. One option for treatment may be a re-sleeve operation. There are inadequate data to properly appraise this option, and further studies must clarify the utility of this procedure in comparison to RYGBP or DS as a second operation.

In addition because of the moderate rate of major complications of 8.0 % (8/100), SG can be recommended as a first-step operation before malabsorptive interventions. Regarding postoperative complications, there were no significant differences among the BMI categories. However, patients with BMI > 60 kg/² required a change to laparotomy significantly more often because of an insufficient intraabdominal view. Preoperative implantation of a gastric balloon to reduce morbidity for patients with BMI > 60 kg/² still needs to be addressed. Especially in patients with BMI above 60 kg/² general complication rate is increasing, due to the fact of an increased pulmonary complication risk, longer operation time and a higher risk for renal complications especially rhabdomyolysis [17].

In this study, there was a 30-day mortality of 0.0 %, a hospitalization mortality of 1.0 %, and a one-year mortality of 2.0 %. There were 2 patients who did not benefit from SG. One patient with a preoperative BMI of 50.5 kg/² first lost weight after SG, but his weight eventually increased to a higher level than before SG (59.7 kg/² by the end of the follow-up). An insufficient change in food patterns and intake of high-calorie foods appeared to be the cause. The other patient, with a preoperative BMI of 55.5 kg/², died after a prolonged course with various complications on day 73 after SG. One other multimorbid patient with a preoperative BMI of 68.0 kg/² died 10 months postoperatively. A causal relationship with SG was excluded after consultation with the family doctor.

The definitive success rate for SG in this study was 98.0 %, with a mortality of 1.0 % and a non-responder rate of 1.0 %. Twenty-five percent of the patients in this study required a second operation via a two-stage procedure for further weight loss.

Nutritional deficits after LSG are rarely evaluated. In postoperative course there is no suggestion for vitamin supplementation. Evidence based data on necessity of supplementation after SG does not exist in literature. After evaluating nutritional deficiencies, there is no need for supplementation after SG, although preoperative existing deficits should be supplemented. Laboratory parameters should be monitored regularly to detect early nutritional deficiencies and to initiate appropriate therapies.

Vitamin B12 levels were in the lower third of the reference range during supplementation. Therefore, it is likely that without supplementation, vitamin B12 deficiencies would have occurred. Therefore, a general vitamin B12 supplementation is advisable to avoid pernicious anemia and to prevent neuropathic pain.

Patients with deficiencies in albumin, vitamin D or calcium have a higher risk of developing osteoporosis; therefore, it is recommended that appropriate supplementations be initiated, even if the concentrations of these parameters are only slightly decreased. PTH levels should be determined to diagnose secondary hyperparathyroidism.

Based on to parameters, iron supplementation should be initiated similar to the supplementation of folic acid. Moreover, supplementation of zinc should be based on symptoms (hair loss, immune deficiency, dry skin). Medication of zinc and calcium should be suggested to intake at different times, because zinc reduces calcium absorption. Supplementation of selenium is not generally necessary because postoperative deficiencies normalize on their own without supplementation, and an adequate, varied food intake seems to be sufficient. Regular determination of laboratory parameters should be performed 6 months after the operation and semiannually thereafter; if the patient’s weight stabilizes, laboratory parameters should be determined once a year.

## Conclusions

Our results following SG and those reported in the literature are promising. Adequate long-term results are still unavailable because long-term studies (> 6 years) are rarely performed. The effectiveness and safety of SG are encouraging.

The operative treatment is not comparable among studies because of a lack of standardization [9]. Also, the 3^rd^ International Consensus Statement on Sleeve Gastrectomy could not recommend which part of the antrum should be left and to what degree the antrum should be minimized to achieve a long-term volume reduction in the sleeve [8]. Evidence-based data are unavailable concerning the size of the bougie or whether the use of staple line reinforcement could reduce the rates of leakage [18].

Our data suggest:

SG is an effective intervention for weight loss. For patients with a BMI of 35–49.9 kg/², a single-step procedure is suitable. For patients with a BMI > 50 kg/², SG is suitable as a first-step procedure for reducing perioperative risks for DS [8; 17].

for patients with BMI > 60 kg/², preoperative implantation of a gastric balloon should be discussed with the aim to reduce morbidity and mortality.

Supplementation of vitamin B12 is indicated and should generally be initiated after SG.

Supplementation of iron and folic acid should depend on laboratory parameters for both genders.

A deficiency in albumin was not reproducible in our patients.

Supplementation of zinc should be based on symptoms.

Substitution of selenium is not necessary.

## Competing interests

The undersigned authors attest that we have no commercial associations (e.g., equity ownership or interest, consultancy, patent and licensing agreements, or institutional and corporate associations) that might present a conflict of interest in relation to the submitted manuscript. (N. Pech on behalf of the co-authors).

## Authors’ contribution

All authors read and approved the final manuscript.

## Pre-publication history

The pre-publication history for this paper can be accessed here:

http://www.biomedcentral.com/1471-2482/12/13/prepub
